# Age-dependent association of white matter abnormality with cognition after TIA or minor stroke

**DOI:** 10.1212/WNL.0000000000007772

**Published:** 2019-07-16

**Authors:** Giovanna Zamboni, Ludovica Griffanti, Sara Mazzucco, Sarah T. Pendlebury, Peter M. Rothwell

**Affiliations:** From the Centre for Prevention of Stroke and Dementia (G.Z., L.G., S.M., S.T.P., P.M.R.) and Wellcome Centre for Integrative Neuroimaging, FMRIB (G.Z., L.G.), Nuffield Department of Clinical Neurosciences, John Radcliffe Hospital, University of Oxford; and Department of Biomedical, Metabolic and Neural Sciences and Centre for Neurosciences and Neurotechnology (G.Z.), University of Modena and Reggio Emilia, Italy.

## Abstract

**Objective:**

To investigate if the association between MRI-detectable white matter hyperintensity (WMH) and cognitive status reported in previous studies persists at older ages (>80 years), when some white matter abnormality is almost universally reported in clinical practice.

**Methods:**

Consecutive eligible patients from a population-based cohort of all TIA/nondisabling stroke (Oxford Vascular Study) underwent multimodal MRI, including fluid-attenuated inversion recovery and diffusion-weighted imaging, allowing automated measurement of WMH volume, mean diffusivity (MD), and fractional anisotropy (FA) in normal-appearing white matter using FSL tools. These measures were related to cognitive status (Montreal Cognitive Assessment) at age ≤80 vs >80 years.

**Results:**

Of 566 patients (mean [range] age 66.7 [20–102] years), 107 were aged >80 years. WMH volumes and MD/FA were strongly associated with cognitive status in patients aged ≤80 years (all *p* < 0.001 for WMH, MD, and FA) but not in patients aged >80 years (not significant for WMH, MD, and FA), with age interactions for WMH volume (*p*_interaction_ = 0.016) and MD (*p*_interaction_ = 0.037). Voxel-wise analyses also showed that lower Montreal Cognitive Assessment scores were associated with frontal WMH in patients ≤80 years, but not >80 years.

**Conclusion:**

MRI markers of white matter damage are strongly related to cognition in patients with TIA/minor stroke at younger ages, but not at age >80 years. Clinicians and patients should not overinterpret the significance of these abnormalities at older ages.

White matter hyperintensity (WMH) of presumed vascular origin detectable on MRI is associated with cognitive impairment and dementia.^1,2^ Associations between cognitive scores and measures of WMH load have been shown in elderly individuals without dementia,^3–6^ patients with manifested arterial disease,^7^ and patients with TIA or minor stroke.^8^ Measures of white matter microstructural integrity estimated using diffusion tensor imaging (DTI), such as fractional anisotropy (FA) and mean diffusivity (MD), are also associated with cognitive deficits in elderly individuals without dementia^9^ including those with small vessel disease.^10,11^ Importantly, it has been shown that these DTI measures are abnormal not only in WMH regions, but also in the surrounding normal-appearing white matter (NAWM), and that the level of DTI-detected deterioration of NAWM is associated with age and WMH burden in cognitively healthy adults,^12,13^ including those older than 90,^14^ as well as poststroke patients.^15,16^ These findings suggest that DTI modifications precede the occurrence of WMH and better capture the true extent of pathophysiologic changes underlying global white matter.^17,18^

The prevalence of WMH increases with age, particularly after age 80,^19,20^ and DTI estimates of white matter integrity also sharply deteriorate with age.^21^ However, there are few data on the age-specific association between MRI-detectable white matter damage and cognition, with most studies reporting associations pooled across a broad range of ages (e.g., ≥50,^7^ >60,^3^ or >65^22^). Yet, some studies suggest that the association may attenuate at age >80,^23,24^ although, to our knowledge, no studies have directly compared the association in older vs younger adults.

Establishing the relevance of WMH to cognition in very old patients is increasingly important because individuals over 80 years of age represent the most rapidly growing segment of the population,^25^ with the greatest concern about risk of dementia.^26–28^ Furthermore, MRI is now very frequently performed as first-line brain imaging for a wide range of neurologic symptoms, such that some WMH are almost always reported in older patients.^19,29^ The most common indication for such imaging in routine practice is after TIA and stroke, and patients frequently have evidence of small vessel disease, inevitably raising concern about vascular cognitive impairment.^30–32^ We therefore studied MRI markers of white matter damage and cognitive status in a population-based cohort of patients with TIA or minor stroke, comparing those aged ≤80 vs >80 years. We also explored with voxel-wise analyses what WMH location is more strongly associated with cognitive impairment and whether there would be differences between age groups.

## Methods

### Study population

Consecutive patients were recruited between March 2012 and June 2016 from the Oxford Vascular Study (OXVASC), a prospective cohort study of all acute vascular events in a defined population of 92,000 residents registered with 100 primary care physicians in Oxfordshire, and the only population-based study of all vascular disease that does not exclude very old patients. After a suspected nondisabling cerebrovascular event (NIH Stroke Scale score <4), OXVASC participants undergo brain MRI, detailed clinical characterization, and cognitive assessment, with face-to-face follow-up at 1, 3, 6, 12, 24, and 60 months. In order to avoid any selection bias, particularly against older patients, patients with a previous TIA or minor stroke prior to the imaging study period were included. Exclusion criteria specific for the purposes of the present imaging study were (1) MRI contraindication or known claustrophobia; (2) intracranial space-occupying lesion; (3) intracranial hemorrhage; (4) brain defect due to previous neurosurgery or developmental anomalies; (4) large chronic, subacute, or acute infarcts (i.e., >2.5 cm on T1-weighted, T2-weighted, or diffusion-weighted imaging sequences); (5) significant movement artefacts that would impair registration; (6) inability to perform cognitive testing (i.e., due to language barriers).

### Standard protocol approvals, registrations, and patient consents

Written informed consent was obtained from all participants. OXVASC was approved by the local ethics committee (Research Ethics Committee reference number: 05/Q1604/70).

## Data availability

Requests for data from the OXVASC Study will be considered by P.M.R. in line with data protection laws. The general policy is that as long as the proposed use of the data is scientifically valid and as long as ethics approval permits, suitably anonymized data can be shared with other researchers.

### Cognitive status

Participants were divided into 3 groups according to their Montreal Cognitive Assessment (MoCA) scores, which has been shown to be sensitive to detect vascular cognitive impairment^33–35^: no cognitive impairment (NoCI, MoCA ≥26), mild cognitive impairment (MildCI, 20 < MoCA < 26), or severe cognitive impairment (SevereCI, MoCA ≤20). These cutoffs were chosen on the basis of previous work showing that the MoCA has high sensitivity in identifying poststroke patients with mild but also severe/multidomain cognitive impairment.^34,36^ For the purpose of the present study, we used MoCA scores from the 1-month follow-up as these better reflect the cognitive status independent from transient cognitive variations related to the minor cerebrovascular event.^37^

### Imaging acquisition

All images were acquired on a 3T Verio (Orem, UT) MRI scanner. The imaging protocol used until December 2014 included fluid-attenuated inversion recovery (FLAIR) (repetition time [TR]/echo time [TE]/inversion time [TI] 9,000/94.0/2,500 ms, flip angle 150°, field of view [FOV] 200 mm, voxel size 0.8 × 0.8 × 5 mm with 1.5 mm interslice gap), post-gadolinium T1-weighted imaging (TR/TE/TI 1,250/4.63/900 ms, flip angle 16°, FOV 220 mm, voxel size 1.1 × 1.1 × 3 mm with 1.5 mm interslice gap), and diffusion-weighted imaging (TR/TE 4,000/106 ms, generalized autocalibrating partial parallel acquisition [GRAPPA] factor 2, FOV 230 mm, voxel size 1.8 × 1.8 × 4 mm with 1.2 mm interslice gap, 12 directions, b value 1,000 s/mm^2^).

The protocol used from January 2015 included high-resolution T1 (TR/TE/TI 2,000/1.94/880 ms, flip angle 8°, FOV 256 mm, voxel size 1 × 1 × 1 mm), FLAIR (TR/TE/TI 9,000/88/2,500 ms, flip angle 150°, FOV 192 mm, voxel size 1 × 1 × 3 mm), and diffusion-weighted imaging (TR/TE = 8,000/86 ms, GRAPPA factor 2, flip angle 16°, FOV 192 mm, voxel size 2 × 2 × 2 mm, 32 directions, b value 1,500 s/mm^2^).

Measures of white matter damage (WMH volumes, MD and FA in NAWM) obtained from the second protocol were standardized on values obtained from the first protocol to allow statistical analyses across the whole sample. In addition, protocol type was added as covariate of no interest on univariate and voxel-wise analyses.

Presence/absence of lacunar infarcts was rated by stroke neurologists and neuroradiologists who were blind to the cognitive scores. Lacunar infarcts were defined as hypointense lesions on T1 imaging with corresponding hyperintense lesion on FLAIR images with a diameter <15 mm.

### WMH measurement

WMHs were automatically segmented on FLAIR images with brain intensity abnormality classification algorithm (BIANCA), a fully automated, supervised method for WMH detection, which gives the probability per voxel of being WMH.^38^ The total WMH volume was calculated from the voxels exceeding a probability of 0.9 (which gave the highest accuracy on this dataset, as tested in our previous work^38^) of being WMH and located within a white matter mask. Obtained values were adjusted for the total brain and ventricles volume (i.e., the sum of the volumes of gray matter, white matter, and ventricles) calculated from the brain-extracted images using FSL’s brain extraction tool (BET)^39^ and log transformed,^40^ as a proxy for intracranial volume that could be obtained from the available FLAIR images.

For voxel-wise analyses, the thresholded and masked WMH maps were binarized and transformed into Montreal Neurological Institute (MNI) standard space, applying the nonlinear registration (FMRIB’s nonlinear image registration tool [FNIRT])^41^ calculated from FLAIR to MNI (via high-resolution T1, if available). We further thresholded the transformed maps at 0.5, binarized them, and applied spatial smoothing of full width at half maximum = 6 mm to compensate for registration errors (the size of the smoothing kernel was empirically decided to be the same as the maximum voxel dimension). The resulting maps were entered into voxel-wise WMH statistical analyses.

WMH were also visually rated on the Fazekas WMH scale allowing categorical measurement of periventricular and deep WMH in grades from 0 (absent) to 3 (severe).^42^

### Measurements of microstructural white matter integrity in NAWM

**Figure 1:**
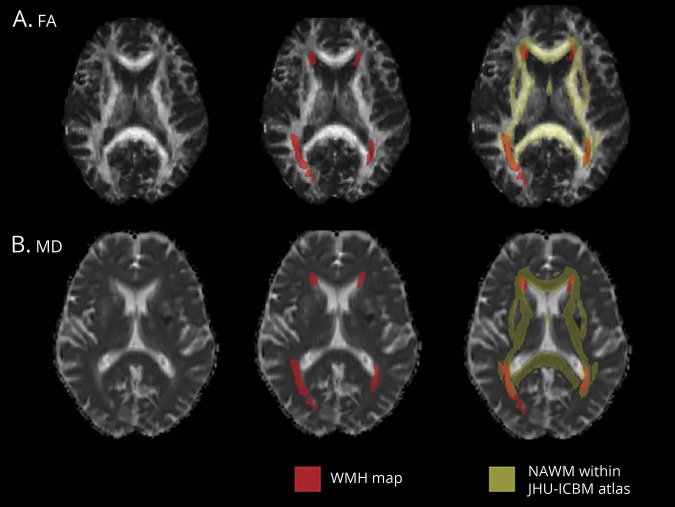
Diffusion tensor imaging (DTI) measurements in normal-appearing white matter (NAWM) Examples of (A) fractional anisotropy (FA) and (B) mean diffusivity (MD) maps registered in Montreal Neurological Institute space from a single participant. Average FA and MD value within (NAWM) was calculated from the regions shown in yellow, that is, within a mask including all the main white matter tracts from the JHU-ICBM DTI atlas and excluding regions of white matter hyperintensity (WMH) obtained with brain intensity abnormality classification algorithm (red).

Diffusion-weighted images were first corrected for head motion and eddy currents. DTI was then performed to create MD and FA maps by fitting a tensor model to the diffusion-weighted images using FMRIB’s Diffusion Toolbox.^43^ Images were brain-extracted using FSL’s BET. All participants' FA maps were then nonlinearly registered to a common diffusion space (FMRIB58_FA, an FA template in MNI space) using FNIRT and the same transformation was applied to MD data. For each participant, we calculated the linear transformation from FLAIR to diffusion data (b = 0 image used as reference) and combined it with the nonlinear transformation from diffusion data to the common space calculated before. The resulting transformation was then applied to WMH maps to register them from FLAIR to diffusion common space. MD and FA values from the NAWM were calculated as average from voxels outside the WMH map, within a mask including all the main white matter tracts in the JHU-ICBM DTI atlas (figure 1). The evaluation of DTI-derived measures was restricted to the main white matter tracts and not performed in the whole NAWM in order to focus on the tracts that are more consistent across participants. This also allows compensating for possible registration errors occurring in the rest of the white matter. In addition, to exclude the possibility of a bias in the results due to a systematic difference in registration quality across age groups, we calculated a cost metric (root mean square difference) between each participant's FA image registered to the template and the template itself and verified that it was not significantly different between age groups (estimated root mean square difference = 0.0420 ± 0.0039; <80 years only = 0.0418 ± 0.0039; >80 years only = 0.0426 ± 0.0041).

### Statistical analyses

Patients were grouped by age ≤80 vs >80 years and cognitive status (NoCI, MildCI, SevereCI, respectively) as defined above. Comparisons between age groups were performed with Mann-Whitney or independent *t* test, as appropriate, for continuous variables, and χ^2^ tests for dichotomous variables using SPSS version 22.0 (SPSS Inc., Chicago, IL). Age-related differences in the associations between MRI markers of white matter damage and cognitive status were studied with 2 × 3 factorial analyses of variance (ANOVAs). Results were considered significant at *p* < 0.05.

Sensitivity analyses were conducted to control for the effects of potential confounders. These analyses included MRI protocol, sex, years of education, head size, presence of lacunes, and number of vascular risks factors (i.e., the sum of hypertension, diabetes mellitus, atrial fibrillation, hyperlipidemia, and smoking). Furthermore, to account for differences in sample size between age groups, the ANOVA was repeated using 4 random subsamples of young participants stratified on cognitive status to match the original large sample.

### Voxel-wise analysis of WMH

We performed the same 2 × 3 factorial ANOVA at the voxel level on the maps of WMH obtained with BIANCA to study the location in the brain of age-by-cognitive status interactions. We then performed correlational voxel-wise analysis on the same maps to test the association between higher probability of having WMH and lower MoCA score in patients aged ≤80 and in patients aged >80, separately for the 2 groups.

All the statistical analyses were performed with nonparametric permutation tests using the randomise tool in FSL,^44^ with protocol as nuisance covariate, and restricted to a white matter mask. Results were considered significant at *p* < 0.05 fully corrected for multiple comparisons using family-wise error correction at the voxel level.^44^

## Results

Among 570 consecutive eligible patients, 4 were excluded due to subsequently diagnosed WMH mimics (multiple sclerosis) and known other causes of dementia (cerebral autosomal dominant arteriopathy with subcortical infarcts and leukoencephalopathy and Alzheimer disease). Table 1 reports the characteristics of the 566 patients included in the WMH analyses, also divided by age groups (i.e., ≤80 and >80). Diffusion-weighted MRI, allowing measurement of FA and MD, were acquired from a subsample of 498 consecutive participants (88%), as this sequence was not initially included in the protocol. Sensitivity analyses on WMH volumes (available on 566 participants) were repeated in the subsample of 498 participants and gave similar results.

**Table 1:**
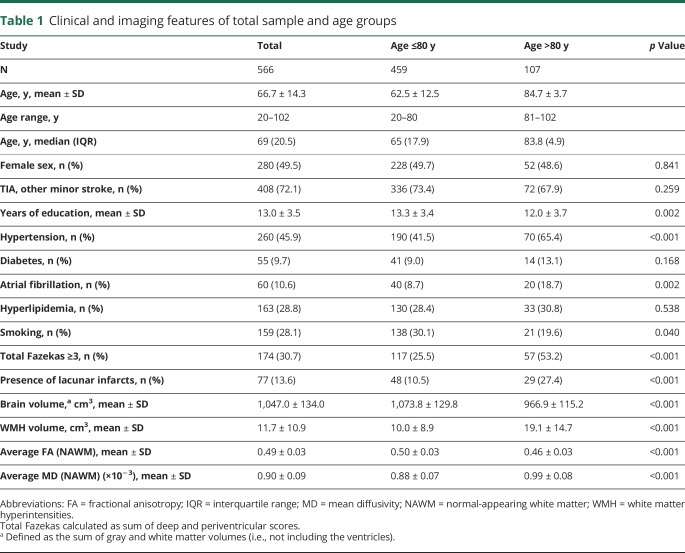
Clinical and imaging features of total sample and age groups

### Age-related associations between WMH volume and cognitive status

**Figure 2:**
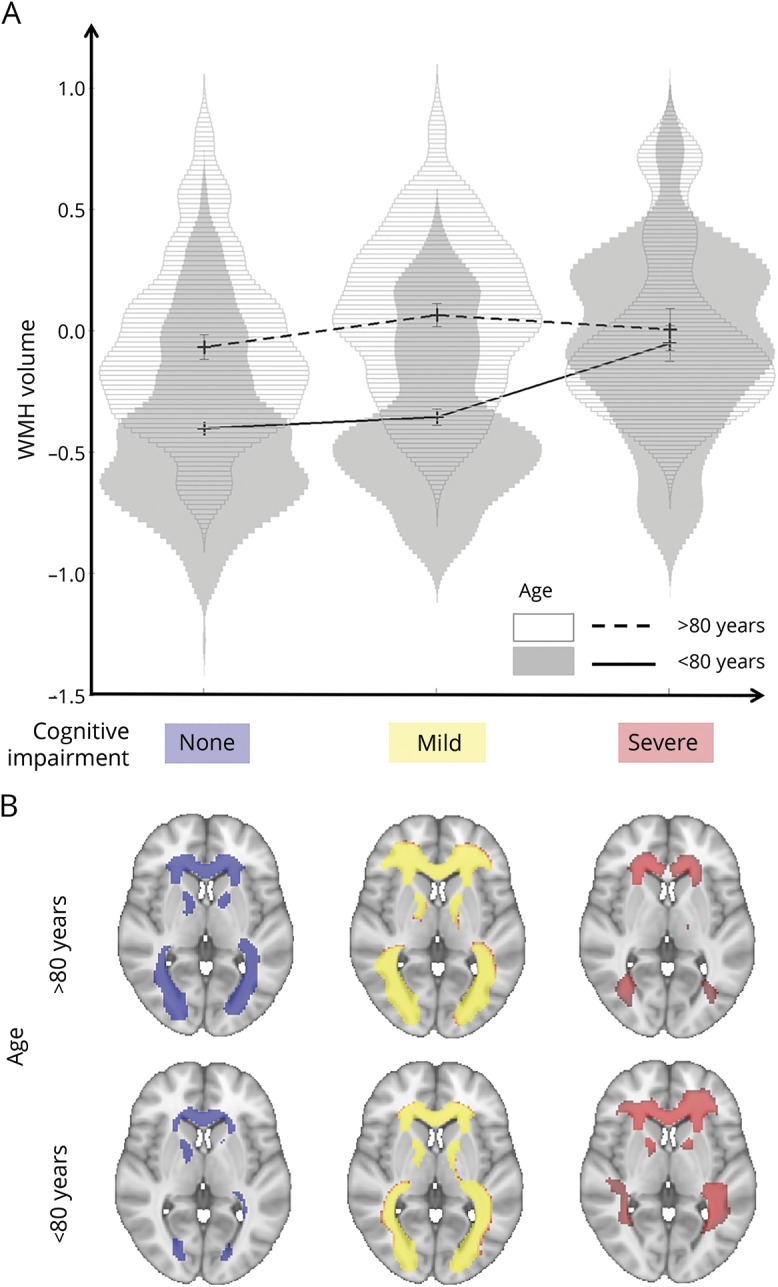
Age-related associations between white matter hyperintensity (WMH) volume and cognitive status (A) Violin plots of the adjusted, log-transformed WMH volumes for the 6 groups of interest obtained by dividing patients according to cognitive status and age. Error bars are ± 1 SD. (B) Average maps of WMH distribution for each group. First row, patients aged >80; second row, patients ≤80. Left: No cognitive impairment (CI). Middle: Mild CI. Right: Severe CI (neurovault.org/collections/2763/).

A 2 × 3 factorial ANOVA on WMH volumes showed a main effect of age (*F* = 34.95, *p* < 0.001), a main effect of cognition (*F* = 6.61, *p* = 0.001), and a significant interaction between age and cognition (*F* = 4.16, *p*_interaction_ = 0.016). The interaction suggested that associations between WMH and cognitive status are different between the age groups (age ≤80 vs > 80 years, figure 2). Follow-up one-way ANOVAs confirmed that there were WMH volume differences across cognitive groups only in patients aged ≤80 and not in patients aged >80 (table 2), even when correcting for within-group age (*F* = 6.17, *p* = 0.002 for age ≤80; *F* = 1.06, *p* = 0.351 for age >80). Sensitivity analyses showed that the interaction remained significant (*p*_interaction_ = 0.046) when adjusting for MRI protocol, sex, years of education, head size, presence of lacunes, and number of vascular risk factors. It also remained significant when repeated using groups of equal sample sizes obtained by randomly splitting the group of young participants in 4 subsets matched on cognition to the original one (table 3).

**Table 2:**
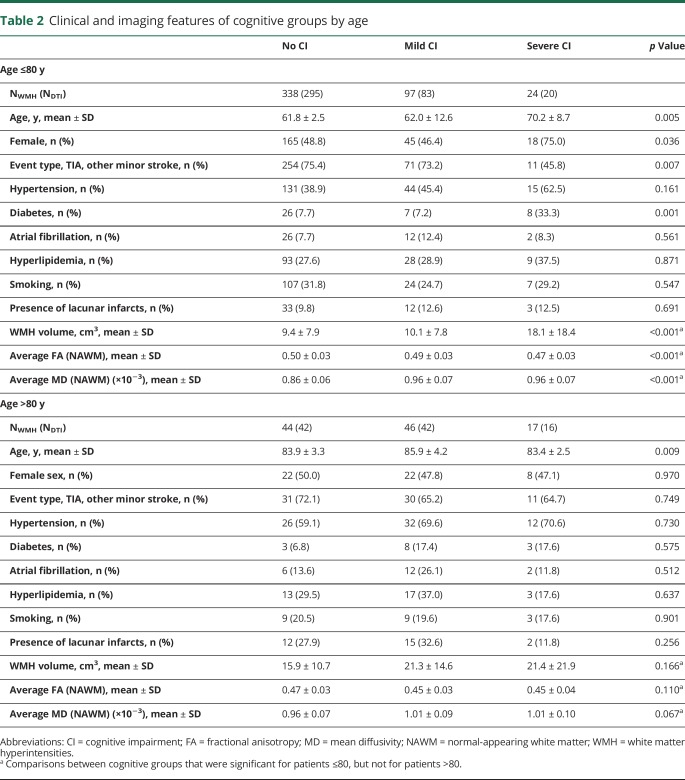
Clinical and imaging features of cognitive groups by age

**Table 3:**
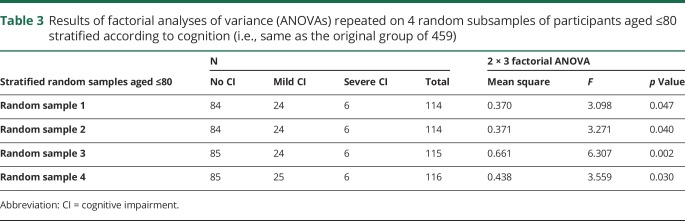
Results of factorial analyses of variance (ANOVAs) repeated on 4 random subsamples of participants aged ≤80 stratified according to cognition (i.e., same as the original group of 459)

The association between high WMH load (above vs below the 80th percentile) and dementia (SevereCI vs NoCI) was strong at age ≤80 (odds ratio [OR] 4.0, 95% confidence interval [CI] 1.65–9.71, *p* = 0.001) but absent at age >80 (OR 0.89, 95% CI 0.26–3.03, *p* = 0.86). These associations were unchanged when the analysis was repeated with the 80th percentile cutoff determined separately in the 2 age groups (age ≤80: OR 3.9, 95% CI 1.68–9.17, *p* = 0.001; age >80: OR 1.6, 95% CI 0.41–6.47, *p* = 0.488).

A 2 × 3 factorial ANOVA with years of education as dependent variable showed no significant interaction between age and cognitive groups (*p* = 0.693), nor did another ANOVA with brain and ventricles volume as dependent variable (*p* = 0.159).

### Age-related associations between MD and FA in NAWM and cognitive status

A 2 × 3 factorial ANOVA on average MD values extracted from NAWM showed a main effect of age (*F* = 73.58, *p* < 0.001), a main effect of cognitive status (*F* = 14.84, *p* < 0.001), and a significant interaction between age and cognitive status (*F* = 3.32, *p*_interaction_ = 0.037). Sensitivity analyses showed that the interaction on MD from NAWM remained significant when adjusting for MRI protocol, sex, years of education, head size, presence of lacunes, and number of vascular risks factors (*p*_interaction_ = 0.048). A similar 2 × 3 factorial ANOVA on average FA values extracted from NAWM showed a main effect of age (*F* = 41.74, *p* < 0.001), and a main effect of cognitive status (*F* = 13.67, *p* < 0.001), but no interaction (*F* = 1.52, *p*_interaction_ = 0.219).

One-way ANOVAs on average MD and FA values extracted from NAWM confirmed that there were differences across cognitive groups only in patients aged ≤80, but not in patients aged >80 (table 2). Adding age or WMH volume as covariates (analyses of covariance) did not change the results (table 4).

**Table 4:**
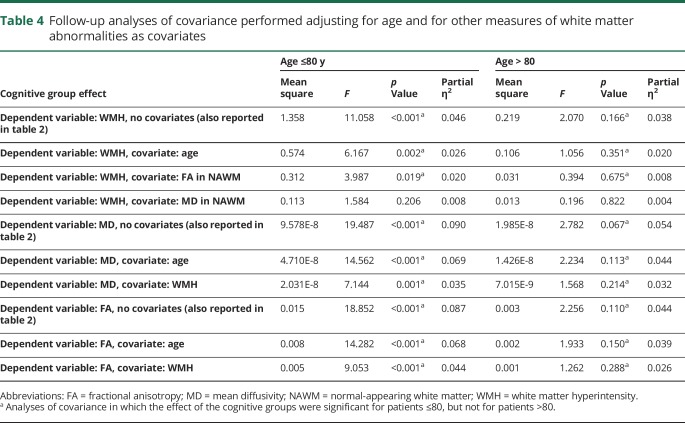
Follow-up analyses of covariance performed adjusting for age and for other measures of white matter abnormalities as covariates

### Localization of WMH relevant to cognitive status

**Figure 3:**
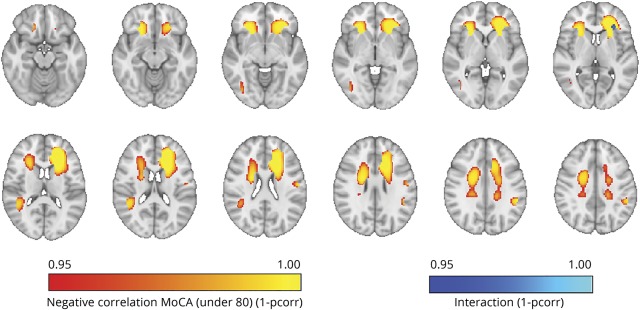
Localization of white matter hyperintensity (WMH) relevant to cognitive status In red–yellow, regions of significant correlation between higher probability of having WMH and lower Montreal Cognitive Assessment scores in patients aged ≤80 years. The same correlational analyses in patients aged >80 years did not lead to significant results. In blue–light blue, region of significant interaction between age and cognitive status resulting from the 2 × 3 voxel-wise analysis of variance (neurovault.org/collections/2763/).

The voxel-wise 2 × 3 factorial ANOVA showed an interaction between cognitive status and age group in an area of the left deep frontal white matter (figure 3, blue–light blue). The voxel-wise correlational analysis performed in patients aged ≤80 showed that in this group the association between the probability of having WMH and lower MoCA score was localized in periventricular frontal and parietal white matter areas bilaterally, more extended on the left hemisphere (figure 3, red–yellow) (see resulting maps on neurovault.org/collections/2763/). The voxel-wise correlational analysis in patients aged >80 instead showed no voxel-wise associations between WMH and MoCA score.

Voxel-wise results did not significantly change when controlling for age within each group.

## Discussion

We found high WMH load in patients with previous TIA or minor stroke aged >80, but showed that, in this age group, it was not significantly associated with cognitive impairment. High WMH load was strongly associated with cognition only in patients aged ≤80, who were 4 times more likely to have severe impairment than patients aged ≤80 with low WMH load. The lack of significant association between WMH and cognition in patients aged >80 also persisted on voxel-wise analyses of WMH distribution, which are expected to be more sensitive than the simple measure of total WMH volume. Finally, we explored if DTI measures of microstructural integrity in NAWM (i.e., outside WMH) would correlate better with cognitive status, but again found no significant associations at age >80.

This loss of association between WMH and cognition at older ages might seem at odds with many previous studies showing significant associations between white matter damage and cognition in the general population or in groups of patients aged >50, 60, or 65.^1–3,7^ However, 2 studies found no associations between WMH and cognition in community-dwelling elderly and stroke survivors older than 80.^23,24^ Our findings, taken together with these 2 previous studies, have important implications for interpretation of brain imaging at older ages. First, MRI has become the recommended first-line imaging investigation for several neurologic conditions affecting older people, and some WMH are almost universally reported in elderly patients. Our results suggest that high WMH loads in patients aged >80 may be considered not excessively concerning by clinicians, patients, or their families. However, the presence of WMH at younger ages should prompt further investigation of possible cognitive impairment. Second, MRI markers of white matter damage have been recommended for use as a proxy of vascular cognitive impairment,^11,45,46^ but interpretation may be more complex in patients aged >80.

Since WMH represents late-stage macroscopic damage of the white matter that occurs as a result of small vessel disease, we also studied microstructural markers of white matter integrity associated with interstitial fluid mobility and water content (namely MD and FA) outside the WMH regions in macroscopically normal-appearing white matter. These measures have been argued to be better markers of cognitive decline in patients with symptomatic cerebrovascular disease and to be more sensitive to change.^11^ Yet we did not find a strong association between these measures and cognitive status in patients >80, suggesting that our findings are not due to intrinsic limitations of the particular MRI marker adopted. Neuropathologic studies also showed that the association between dementia and postmortem evidence of vascular pathology attenuates in the very old.^47^

The lack of association between white matter abnormality and cognitive impairment in patients >80 was mainly driven by patients with substantial white matter disease and normal cognition. Patients aged ≤80 with similar degree of damage instead showed severe cognitive impairment. This finding goes against the hypothesis that a certain threshold of WMH is needed to affect cognition.^23^ It also suggests that the lack of correlation between WMH and cognition in patients aged >80 could not simply be due to the fact that patients with severe white matter damage might have already died by the age of 80, as if this were the case the group aged >80 would be expected to have little white matter abnormality. However, WMH may have diverse underlying pathologies, some not affecting cognition or life expectancy, others increasing susceptibility to dementia and death (i.e., only patients whose white matter pathology caused dementia had died by the age of 80). In addition, since we studied all consecutive patients presenting to a TIA/stroke clinic, we cannot exclude the possibility of a sampling bias due to the fact that elderly patients with severe cognitive impairment or dementia may not present to medical attention for a suspected TIA. However, previous studies on community-dwelling elderly not subject to presentation bias similarly found no association between white matter abnormalities and cognition.^14,23^ Yet the clinical implications of our study (i.e., the lack of association between WMH and cognitive impairment in patients aged >80) remain, irrespective of the mechanism, as—ultimately—the group of patients relevant to clinicians only includes those who present to medical attention. Of note, the lack of correlation between WMH and cognition in patients aged >80 parallels the tendency for the associations between other risk factors and dementia to attenuate with advancing ages.^48^

One of the strengths of our study is that it addresses a major limiting factor in non-population-based studies by not excluding very old patients. In addition, we imaged a relatively homogeneous population in that all patients had recent symptomatic cerebrovascular disease, such that confounding by greater comorbidities or vascular risk factors in the older age group is less likely, as also shown by sensitivity analyses including potential confounders such as brain size, education, number of vascular risks factors, and presence of lacunar infarcts as covariates.

Several limitations should be highlighted. First, we looked only at the cross-sectional associations with cognitive status and cannot be certain that associations would be similar on long-term follow-up. Second, we used only the MoCA as a screening tool for cognitive impairment, and so we cannot exclude subtler cognitive deficits in older patients with severe white matter disease, which might have been evident on more detailed neuropsychological assessment. However, we were primarily interested in clinically overt cognitive impairment. Third, we did not adjust for the presence of subclinical depression or other neuropsychiatric disorders, which may affect cognitive performance. However, we would expect the effect of stroke-related depression to be similar across the 2 age groups,^49^ as these did not differ in the severity of cerebrovascular event. Finally, we cannot exclude that the lack of correlation between WMH and cognition in patients aged >80 was merely due to differences in sample size. However, we believe this possibility to be extremely unlikely considering that we found a significant interaction and that our results agree with other findings in the literature.^14,23,50^ Future studies with larger sample sizes in patients older than 80 are required to support the identified lack of clinical significance of MRI markers.

Our findings confirm the association between MRI markers of white matter damage and cognitive impairment in patients younger than 80. They also suggest that the clinical significance of MRI markers might not be overinterpreted in patients older than 80, or considered a good proxy of vascular cognitive impairment in trials or research studies on this age group.

## Glossary

ANOVA: analysis of variance
BET: brain extraction tool
BIANCA: brain intensity abnormality classification algorithm
CI: confidence interval
DTI: diffusion tensor imaging
FA: fractional anisotropy
FLAIR: fluid-attenuated inversion recovery
FNIRT: FMRIB’s nonlinear image registration tool
FOV: field of view
GRAPPA: generalized autocalibrating partial parallel acquisition
MD: mean diffusivity
MildCI: mild cognitive impairment
MNI: Montreal Neurological Institute
MoCA: Montreal Cognitive Assessment
NAWM: normal-appearing white matter
NoCI: no cognitive impairment
OR: odds ratio
OXVASC: Oxford Vascular Study
SevereCI: severe cognitive impairment
TE: echo time
TI: inversion time
TR: repetition time
WMH: white matter hyperintensity
